# Do people with cognitive impairment benefit from cochlear implants? A scoping review

**DOI:** 10.1007/s00405-024-08719-5

**Published:** 2024-06-07

**Authors:** Piers Dawes, Hannah Cross, Rebecca Millman, Iracema Leroi, Christiane Völter

**Affiliations:** 1 School of Health and Rehabilitation Sciences, Centre for Hearing Research (CHEAR), University of Queensland, Brisbane, QLD Australia; 2 Manchester Centre for Audiology and Deafness, University of Manchester, Manchester, UK; 3 Global Brain Health Institute, Trinity College Dublin, Dublin, Ireland; 4 Cochlear Implant Center Ruhrgebiet, St. Elisabeth Hospital, Ruhr University, Bochum, Germany

**Keywords:** Cochlear implant, Dementia, Mild cognitive impairment

## Abstract

**Purpose:**

To identify and evaluate the evidence for the benefits of cochlear implants for people with cognitive impairment or dementia in terms of speech recognition, quality of life, behavioural and psychological symptoms of dementia, cognition, function in daily life, mental well-being, and caregiver burden.

**Methods:**

Ten electronic databases were searched systematically from inception to December 2023 for studies reporting on outcomes for cochlear implants that included adults identified with cognitive impairment, mild cognitive impairment, or dementia.

**Results:**

Thirteen studies were included in this review with a combined total of 222 cochlear implant patients with cognitive impairment, mild cognitive impairment. Two studies were non-randomised controlled design, the remainder were single group studies, case series or single case studies. Evidence suggested that people with cognitive impairment benefit in terms of improved speech recognition from cochlear implants, although they may benefit less than those with healthy cognition and the degree of benefit depends on the level of cognitive impairment. There was no evidence for increased adverse events among those with cognitive impairment. There was limited or no evidence for any other outcome.

**Conclusion:**

People with cognitive impairment or dementia do benefit from cochlear implants. To inform policy and clinical practice, further data are needed about the broader benefits of cochlear implants for people with cognitive impairment or dementia, and referral, eligibility, and cochlear implant support needs for people with cognitive impairment and their caregivers.

## Introduction

Based on the population estimates of the prevalence and demographics of hearing loss and cognitive impairment, one would predict that around half of adults with severe or profound hearing loss have either mild cognitive impairment or dementia.

Both hearing loss and cognitive impairment are increasingly prevalent with older age. Global prevalence of dementia was estimated at 6.5% among those aged 40–84 years, increasing to 23.5% among those aged over 85 [1]. Prevalence of mild cognitive impairment—a dementia prodrome—was estimated at around 27.8% of people aged over 80 years although estimates vary according to population and study type [2]. In relation to hearing loss, a large US national population survey identified few people with severe or profound hearing loss (≥ 65 dB HL at 0.5–4 kHz) among those aged under 50 years, although prevalence rapidly increased after age 50, up to 7.53% of people aged over 80 years [3]. The greatest numbers of people with severe or profound hearing loss are in older age groups in which dementia and mild cognitive impairment are also most prevalent. Given that hearing loss is associated with lower cognitive performance, increased cognitive decline, and increased dementia risk [4], the actual numbers of people living with both dementia and severe or greater hearing loss is probably greater than would be expected if hearing loss and dementia occurred independently.

With aging populations and increasing numbers of people living with dementia, optimising quality of life for people with dementia is a global priority [5]. Hearing interventions may offer an effective, low-risk, acceptable and desired non-pharmacological solution to improve outcomes for people with dementia or mild cognitive impairment [6, 7]. For cognitively healthy adults, cochlear implants offer improved environmental sound and speech perception, with benefits for quality of life, mental well-being, and social engagement [8–10]. Cochlear implant use is cognitively demanding. Optimal use of cochlear implants requires a period of perceptual learning and adjustment to the stimulus provided by the implant. Cochlear implants also require regular maintenance and incorporation into daily routines. Because of the cognitive demands of cochlear implant use, some may assume that people with cognitive impairments may not be good candidates for cochlear implants or may experience limited benefits from cochlear implants. To our knowledge, the potential benefits and disadvantages of cochlear implants for people with cognitive impairment have not been systematically reviewed.

The objective of this scoping review was to synthesise the evidence base regarding the outcomes of cochlear implants for people with severe or greater hearing loss and cognitive impairment (mild cognitive impairment or dementia) in terms of the following outcomes: (i) adverse events including rates of non-use of cochlear implants, (ii) speech recognition, (iii) hearing-related quality of life, (iv) general quality of life, (v) cognition, (vi) rate of cognitive decline, (vii) behavioural and psychological symptoms of dementia, (viii) mental well-being, (ix) activities of daily living; and (x) caregiver burden.

## Methods

The protocol for this study was published in the Figshare data registry (22584391). Due to the lack of randomised controlled trials in this area, scoping review methodology was selected as the most appropriate means of analysis. Systematically conducted scoping reviews encompass various study designs and broader subject material than systematic reviews [11]. Scoping reviews explore the breadth and depth of the existing research to identify areas requiring further investigation [12]. Acquisition, extraction, assessment and reporting of the data in the present review was carried out according to the Preferred Reporting Items for Systematic Reviews and Meta-Analysis (PRISMA) Statement [13].

### Study selection

Studies were eligible for inclusion if they included adult participants who were: (i) resident in community or long-term care settings; (ii) aged over 50 years (to differentiate from atypical young onset dementia) and (iii) clinically diagnosed with a progressive neurodegenerative condition leading to dementia, including ‘mild cognitive impairment’ (MCI; defined according to Petersen criteria) [14] or minor neurocognitive disorder (according to DSM-5 criteria), ‘dementia’ (National Institute of Neurological and Communicative Disorders and Stroke criteria) or major cognitive disorder (according to DSM-5 criteria) including Parkinson’s disease dementia, frontotemporal dementia, Lewy body disease, vascular dementia and Alzheimer disease or dementia due to other causes. In a change from the registered review protocol, we also included studies with participants identified with cognitive impairment based on a cognitive screening test at study baseline. Participants must have had acquired adult-onset severe or profound neurosensory hearing loss, (determined by audiological testing; e.g. hearing levels over 65 dB HL; at 0.5–4 kHz). Randomised controlled trials, quasi-experimental studies and observational studies were included. For those studies with comparison conditions, the comparison could have included placebo/sham, standard care, alternative intervention, or no intervention.

Outcome measures of interest were: (i) adverse events including rates of non-use of cochlear implants (ii) speech recognition (e.g. consonant-nucleus-consonant (CNC) percent correct word recognition), (iii) hearing-related quality of life (measured with standardised assessments of hearing disability, (e.g. Hearing Handicap Inventory for the Elderly [15]), (iv) general quality of life (measured with standardised assessments, e.g. Health Utilities Index-3 [16]), (v) cognition (as measured with standardised assessments), (vi) rate of cognitive decline (defined as change in cognitive performance), (vii) behavioural and psychological symptoms of dementia (BPSD;^1^ including agitation, aggression, psychosis and apathy; measured with standardised assessments), (viii) mental well-being, (ix) activities of daily living (including both instrumental activities and activities of daily living measured with standardised checklists); and (x) caregiver burden (measured with standardised assessments, e.g. Zarit Burden Interview [17]). Studies were included if published in any language. Both peer reviewed studies and articles from the grey literature were included. Editorials, newspaper articles and other forms of popular media were excluded. Studies were not selected based on methodological quality.

### Data sources

The search strategy included: (i) computer searches of electronic databased; (ii) consultation with an expert network; and (iii) hand-searching the reference lists of eligible papers for additional studies.

The computer search was carried out with Google (where the first 100 results were screened) and Google Scholar, PubMed, Cochrane Central Register of Controlled Trials (CENTRAL), PsycINFO, CINAHL, AgeInfo, Web of Science, and clinical trials registers ClinicalTrials.gov and the WHO international clinical trials registry platform (ICTRP). Search terms were: ("Dementia"[Mesh] OR "Cognitive Dysfunction"[Mesh] OR dementia[tiab] OR alzheimer*[tiab] OR "cognitive impair*"[tiab] OR "cognitive dysfunction"[tiab] OR "cognitive disorder*"[tiab]) AND ("Cochlear Implants"[Mesh] OR "Cochlear Implantation"[Mesh] OR "cochlear prosthes*"[tiab] OR "cochlear implant*"[tiab]).

The computer search was carried out during December 2023. There was no restriction on the publication date; the search period included the earliest possible date in available each database until the date of the search.

To ensure that all available relevant studies were captured in our review, we undertook an international consultation with ten clinical and/or academic experts in audiology and otolaryngology in the UK, Australia, and Germany. The experts were contacted via email and asked whether they were aware of any relevant published, unpublished, or on-going studies that were not identified based on the computer search. Finally, the reference lists of all papers eligible for inclusion in the review were hand searched to identify further studies of interest.

### Study selection

**Fig. 1:**
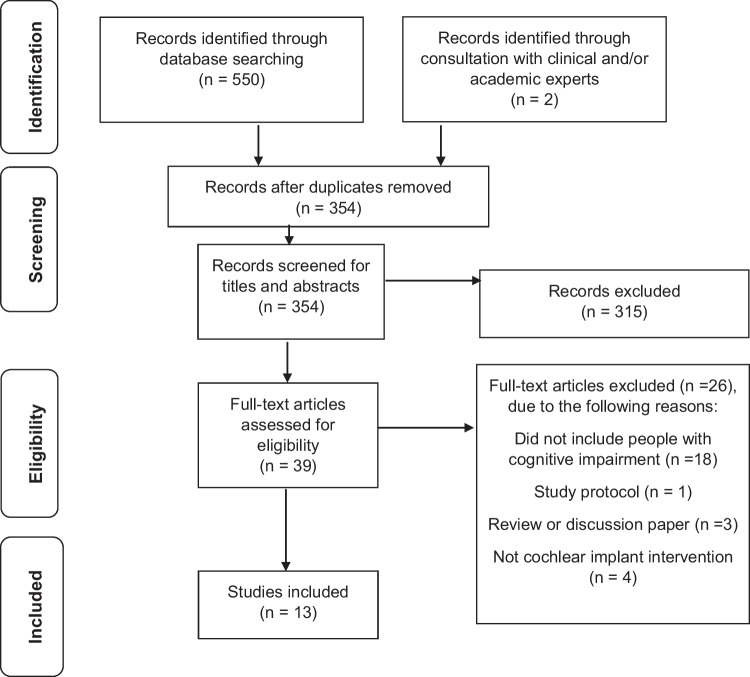
Preferred reporting items for systematic reviews and meta-analyses flow diagram

*Step 1*: Study titles were independently reviewed by the authors and selected for further review if the title included mention of evaluation of cochlear implant intervention or management in adults. *Step 2*: Abstracts were independently reviewed by the first and second authors (PD and HC, k = 0.7 substantial agreement) and selected for further review based on the inclusion criteria described above. If a consensus was not reached, the full text of the paper was reviewed. *Step 3*: Full-text articles of abstracts selected in *Step 2* were reviewed by the first and second authors (k = 0.6 substantial agreement). Disagreements were discussed with additional authors (k = 1.0 perfect agreement following discussion). Full-text articles were reviewed according to the eligibility criteria described above. The study selection process and reasons for exclusion were recorded (Fig. 1).

### Data extraction and analysis

Data extraction was based on the parameters listed in Table 1, including participant details, intervention type, and outcome measure. Data were extracted from the full-text article by one reviewer and checked by a second reviewer. Disagreements were recorded and resolved by involvement of an additional reviewer.

**Table 1:**
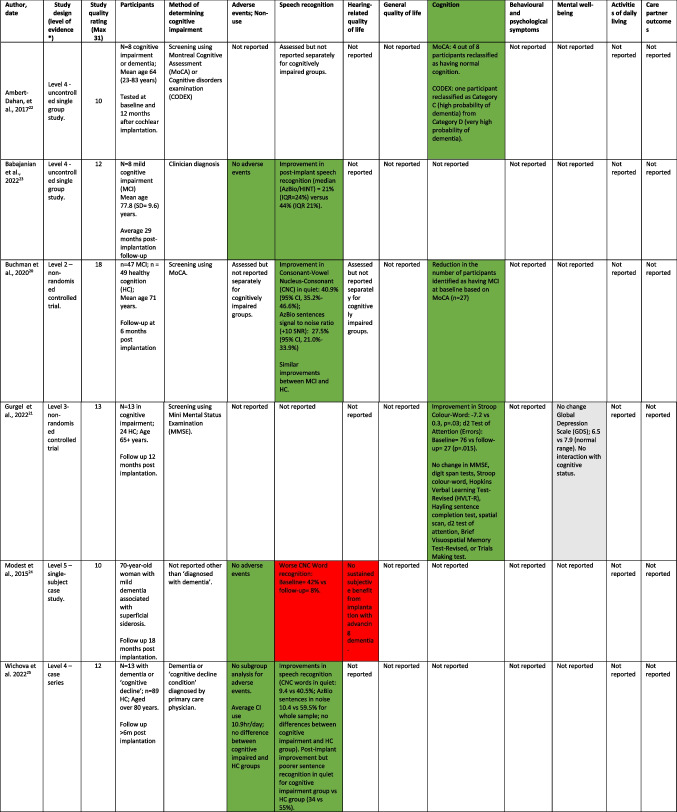

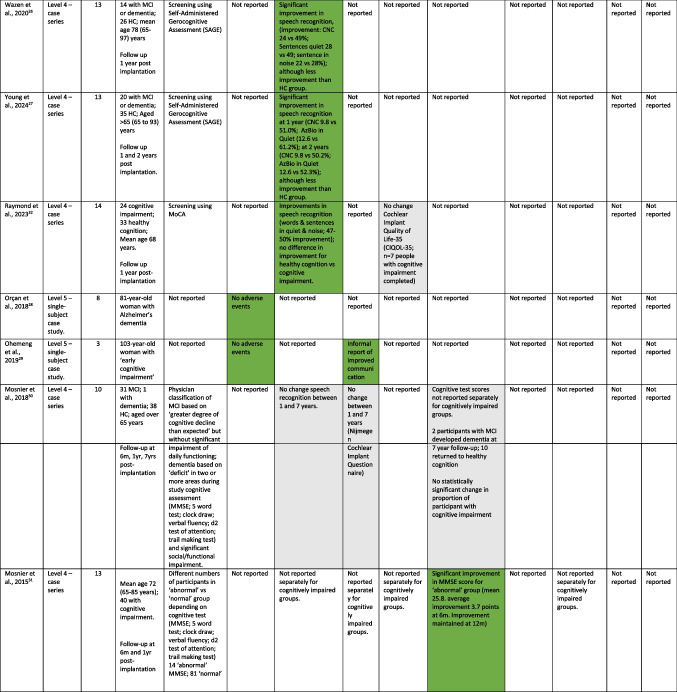
Summary of studies included in the review

Red: worse outcome; Green: improved outcome; Grey: no change

*According to *Oxford 2011 Levels of Evidence* [18]

Results were summarised descriptively according to whether there was a reported improvement, deterioration, or no change in each respective outcome measure. Where the necessary statistics were reported, effect sizes (Cohen’s *d*) for statistically significant changes were calculated with a view to summarise the effects of cochlear implants for each outcome variable, if sufficient data were available. Heterogeneity of study design and outcomes of interest, use of informal outcome measures and lack of reporting of the required statistics precluded statistical synthesis of results across studies. Study design was described according to the *Oxford 2011 Levels of Evidence* [18], which are: Level 1, fully powered randomised controlled trials or meta-analysis; Level 2, controlled trials without randomisation; Level 3, retrospective cohort or case–control studies; Level 4, case series or uncontrolled single group study; and Level 5, expert opinion or case report. Risk of bias was assessed using Downs and Black’s checklist [19] for assessing the quality of randomised and non-randomised studies. The checklist contains 27 items pertaining to the quality of reporting, internal and external validity, and statistical power with a maximum possible score of 31. Study quality ratings were carried out independently by reviewers PD and HC. Any disagreements were recorded and resolved by involvement of an additional reviewer.

## Results

As summarised in Table 1, we identified 13 papers including two non-randomised controlled trials [20, 21], the remainder being uncontrolled single group, case series or case study designs [22–32]. Four studies reported outcomes in the same patient cohort at different time points [26, 27, 30, 31]. Study quality ratings ranged between 3 and 18 out of a maximum of 31 [19]. The main reasons for low quality ratings were the lack of controlled design elements, unclear generalisability to the population of interest and potential for inclusion bias.

Dementia or MCI status was established predominantly based on a cognitive screening test (Mini Mental Status Examination, Montreal Cognitive Assessment, Self-Administered Gerocognitive Assessment, or Cognitive Disorders Examination) either alone or in combination with other cognitive assessments. Four studies mentioned diagnosis by a clinician, although did not provide details [23–25, 30]. Two studies did not provide details concerning diagnosis of cognitive impairment [28, 29].

Most (8 studies) either did not report on adverse events or non-use or did not report data separately for those with cognitive impairment. The remaining 5 studies reported no adverse events [23, 24, 28, 29], or rates similar to those with healthy cognition [25].

Speech recognition was the most frequently assessed outcome measure. Two studies reported post-implantation improvements in speech recognition that were comparable between those with mild cognitive impairment and those with healthy cognition [20, 32]. A further three studies reported less improvement among those with dementia, mild cognitive impairment or ‘cognitive decline condition’ than those with healthy cognition [25–27]. Two studies reported stable benefits in speech recognition at 2 years [27] and 7 years [30] post implant among those with mild cognitive impairment or dementia. In contrast, one single case study reported no long-term benefit in speech recognition from the cochlear implant due to cognitive deterioration in a person with dementia [24].

With respect to patient-reported hearing-related quality of life, or general quality of life, one study reported no change in general quality of life post-implant amongst a small group of people with cognitive impairment [32]. One study contained informal reports of improved communication for a single case study of a person with ‘early cognitive impairment’ [29].

Five studies reported on cognitive outcomes. Four studies reported improvements in cognitive test performance on the Montreal Cognitive Assessment, Cognitive Disorders Examination, Stroop, Test of Attention and Mini Mental Status Examination [20–22, 31], with some people previously identified as having a cognitive impairment reclassified as having healthy cognition based on improved performance on a cognitive screening test [20, 22]. Mosnier et al. [30] reported no statistically significant change in the proportion of people with cognitive impairment after a 7 year follow-up period.

Only one study reported mental well-being outcomes: Gergel et al. [21] reported no statistically significant change in depression symptoms at 12 months post-implant among those with cognitive impairment. Depression symptoms were within the normal range at pre- and post-implant time points.

No studies reported on behavioural and psychological symptoms of dementia, activities of daily living or care partner outcomes.

## Discussion

Cognitive impairment (dementia or mild cognitive impairment) probably affects around half of adults with severe or profound hearing loss who may be candidates for a cochlear implant. To our knowledge, this is the first systematically conducted review of the benefits of cochlear implants for people with cognitive impairment.

### Speech recognition

The weight of evidence suggests that people with dementia or mild cognitive impairment do benefit from cochlear implants with respect to improved speech recognition. Although, perhaps due to the impact of cognitive impairment, improvements in speech recognition may not be as great as for those with healthy cognition on average. Benefits of cochlear implants—including long-term prospects for improved communication—may depend on the severity and progression of cognitive impairment. In Wazen et al.’s analysis [26], there was a weak association (*r*’s 0.15–0.19) between pre-operative cognitive performance and post-operative improvement in speech recognition among people with mild cognitive impairment or dementia. One might predict that people with cognitive impairment may require more time and/or training to optimise speech perception with the implant. From the reports in this review, it was not possible to tell whether post-implant improvements in speech perception occurred more slowly in among those with cognitive impairment versus those with healthy cognition.

People with dementia may require additional support to engage with hearing interventions and obtain optimal benefit from treatment [33–35]. Papers in this review did not report what post-implant rehabilitation regimens were provided to patients, nor whether those with cognitive impairment received additional support. To optimise patient outcomes, cochlear implant clinics should develop robust means of identifying those with cognitive impairment and tailoring post-implant rehabilitation support systems to support the needs of those with cognitive impairment.

### Cognitive outcomes

Perhaps because of the attention devoted to the potential for hearing interventions to prevent dementia [36], several studies reported on cognitive outcomes post implantation. The general pattern was for improvement, although because there were only two studies that included a control group, it is difficult to be sure whether these apparent improvements in cognitive test performance are due to the cochlear implant, due to a retest effect, or simply due to better audibility of cognitive tests with a spoken component. Note that the potential for hearing interventions for improving cognition and preventing dementia is a different issue to improving outcomes for people living with dementia [37].

### Adverse events and non-use

Accelerated cognitive decline has been reported following surgery and general anesthesia [38], along with post-operative cognitive disturbances [39], with adverse outcomes more likely for those with worse pre-operative cognition. In this review, however, there was no suggestion of increased rates of adverse events for those with cognitive impairment. This may be because adverse post-operative events depend on the type of surgery, and cochlear implantation has lower rates of adverse events than other surgeries [40]. Similarly, there was no indication of higher rates of non-use of cochlear implants among people with cognitive impairment compared to those with healthy cognition. One limitation is that non-use of cochlear implants increases over time [8]; most studies in this review included follow-up of up to 1 year only, so any problems with non-use may not have been observed.

### Limitations on current knowledge

A general limitation of the included literature is that studies mostly set out to examine outcomes of cochlear implants for older people in general, rather than people with cognitive impairment in particular. Only 3 of the 13 studies in this review aimed to examine outcomes of cochlear implants for people with cognitive impairment. Because studies generally did not focus specifically on outcomes for people with cognitive impairment, several studies did not report some outcomes separately for those with cognitive impairment versus those with healthy cognition, therefore we were unable to report on potential differences between these groups.

All studies were based on routinely collected clinical data. The limitations of the resulting uncontrolled single group, case series and case study designs make it difficult to establish the benefits of cochlear implants reliably in the face of confounding factors including re-test effects and progressive cognitive decline. Furthermore, a critical limitation was that most studies identified cognitive impairment based on a cognitive screening test e.g., the Mini Mental Status Examination. Some cognitive screening tests are known to be impacted by hearing loss [41], and it was not clear what procedures were used to take this into account. Those studies that did report a clinician diagnosis of cognitive impairment did not supply information about what the diagnosis entailed; sensory impairments may increase likelihood of dementia diagnosis due to impact on cognitive tests and similarity of symptoms of hearing loss and cognitive impairment [42]. In summary, one cannot be sure that the people identified with cognitive impairment in the studies included in this review truly had cognitive impairment. Future research would ideally utilise and report clinical criteria for diagnosis of cognitive impairment or use screening tests for cognitive impairment that have been validated for use with people with hearing loss [43, 44].

The current literature is silent on matters of high clinical relevance, including referral of people with cognitive impairment for cochlear implant evaluation, the type and severity of dementia that would indicate that a person is unlikely to use and benefit from a cochlear implant, and additional support needs of people with dementia and their caregivers. Experience in the case of hearing aid rehabilitation suggests that people with dementia may require additional support to consistently use and obtain optimal benefit from a hearing device [33–35].

It is encouraging that there do appear to be benefits in improved speech recognition for cochlear implants for people with cognitive impairment. Treating hearing loss may be particularly important in the context of dementia, because hearing loss exacerbates the impact of cognitive impairment [6]. Hearing interventions may offer an effective solution for reducing the impact of dementia that is highly desired by people living with dementia and hearing loss [45]. Improving outcomes for people living with dementia is a global priority [5], and there is potential for hearing interventions to improve quality of life, mental well-being, behavioural and psychological symptoms of dementia, functional abilities and carer outcomes [7]. Unfortunately, it was unclear whether the reported improvements in psychometric tests of speech recognition translated into improvements in these other outcomes; other outcomes were mostly not assessed or reported in the included studies. To inform policy and clinical practice, it would be useful to index other outcomes that may be important in the context of cognitive impairment, including quality of life, mental well-being, behavioural and psychological symptoms of dementia, functional abilities, and carer outcomes.

## Conclusions

Based on population data, one would expect that more than half of adults with severe or greater hearing loss live with mild cognitive impairment or dementia. Cochlear implants offer an opportunity to address the global challenge for improving outcomes for people living with dementia. People with mild cognitive impairment or dementia and severe or profound hearing loss do benefit from cochlear implants in terms of improved speech recognition, with no indication of increased rates of adverse events.

## Data Availability

Not applicable.

## Footnotes

1 “Behavioural and psychological symptoms of dementia (BPSD)” and “challenging behavior” are commonly used terms to describe repetitive behaviour, wandering, delusions, hallucinations, sleep disturbances, social inappropriateness, depression/anxiety, and physical aggression among people with dementia. We recognise that ‘BPSD’ is problematic because such terminology objectifies people’s experience. Alternative terminology has been suggested (e.g. “responsive behaviours”, to recognise behaviours have meaning, and there are physical, emotional or physical environmental factors that influence behaviour). As these is no consensus for suitable alternative terminology, we use “behavioural and psychological symptoms of dementia” as it is currently the most used term.

## References

[1] Nichols E et al (2022) Estimation of the global prevalence of dementia in 2019 and forecasted prevalence in 2050: an analysis for the Global Burden of Disease Study 2019. Lancet Public Health 7(2):e105–e125 34998485 10.1016/S2468-2667(21)00249-8 PMC8810394

[2] Overton M, Pihlsgård M, Elmståhl S (2019) Prevalence and incidence of mild cognitive impairment across subtypes, age, and sex. Dement Geriatr Cogn Disord 47(4–6):219–232 31311017 10.1159/000499763

[3] Goman AM, Lin FR (2016) Prevalence of hearing loss by severity in the United States. Am J Public Health 106(10):1820–1822 27552261 10.2105/AJPH.2016.303299 PMC5024365

[4] Dawes P, Völter C (2023) Do hearing loss interventions prevent dementia? Z Gerontol Geriatr 56:1–7 10.1007/s00391-023-02178-z PMC10289956 37140632

[5] Livingston G et al (2020) Dementia prevention, intervention, and care: 2020 report of the Lancet Commission. Lancet 396(10248):413–446 32738937 10.1016/S0140-6736(20)30367-6 PMC7392084

[6] Dawes P et al (2021) Hearing assessment and rehabilitation for people living with dementia. Ear Hear 43(4):1089–1102 34966160 10.1097/AUD.0000000000001174 PMC9197139

[7] Dawes P et al (2019) Interventions for hearing and vision impairment to improve outcomes for people with dementia: a scoping review. Int Psychogeriatr 31(2):203–221 30244688 10.1017/S1041610218000728

[8] Boisvert I et al (2020) Cochlear implantation outcomes in adults: a scoping review. PLoS ONE 15(5):e0232421 32369519 10.1371/journal.pone.0232421 PMC7199932

[9] Bekele Okuba T et al (2023) Cochlear implantation impact on health service utilisation and social outcomes: a systematic review. BMC Health Serv Res 23(1):929 37649056 10.1186/s12913-023-09900-y PMC10468908

[10] Mäki-Torkko EM et al (2015) From isolation and dependence to autonomy–expectations before and experiences after cochlear implantation in adult cochlear implant users and their significant others. Disabil Rehabil 37(6):541–547 24989065 10.3109/09638288.2014.935490

[11] Arksey H, O’Malley L (2005) Scoping studies: towards a methodological framework. Int J Soc Res Methodol 8(1):19–32 10.1080/1364557032000119616

[12] Davis K, Drey N, Gould D (2009) What are scoping studies? A review of the nursing literature. Int J Nurs Stud 46(10):1386–1400 19328488 10.1016/j.ijnurstu.2009.02.010

[13] Moher D et al (2009) Preferred reporting items for systematic reviews and meta-analyses: the PRISMA statement. PLoS Med 6(7):e1000097 19621072 10.1371/journal.pmed.1000097 PMC2707599

[14] Petersen RC (2011) Mild cognitive impairment. N Engl J Med 364(23):2227–2234 21651394 10.1056/NEJMcp0910237

[15] Ventry IM, Weinstein BE (1982) The hearing handicap inventory for the elderly: a new tool. Ear Hear 3(3):128–134 7095321 10.1097/00003446-198205000-00006

[16] Furlong WJ et al (2001) The Health Utilities Index (HUI®) system for assessing health-related quality of life in clinical studies. Ann Med 33(5):375–384 11491197 10.3109/07853890109002092

[17] Zarit SH, Orr NK, Zarit JM (1985) The hidden victims of Alzheimer’s disease: families under stress. NYU Press, New York

[18] OCEBM Levels of Evidence Working Group (2011) The Oxford 2011 levels of evidence. http://www.cebm.net/index.aspx?o=5653. Accessed 5 June 2017

[19] Downs SH, Black N (1998) The feasibility of creating a checklist for the assessment of the methodological quality both of randomised and non-randomised studies of health care interventions. J Epidemiol Community Health 52(6):377–384 9764259 10.1136/jech.52.6.377 PMC1756728

[20] Buchman CA et al (2020) Assessment of speech understanding after cochlear implantation in adult hearing aid users: a nonrandomized controlled trial. JAMA Otolaryngol-Head Neck Surg 146(10):916–924 32857113 10.1001/jamaoto.2020.1584 PMC7453346

[21] Gurgel RK et al (2022) Evaluating the impact of cochlear implantation on cognitive function in older adults. Laryngoscope 132:S1–S15 34738240 10.1002/lary.29933 PMC8920765

[22] Ambert-Dahan E et al (2017) Cognitive evaluation of cochlear implanted adults using CODEX and MoCA screening tests. Otol Neurotol 38(8):e282–e284 28806339 10.1097/MAO.0000000000001464

[23] Babajanian EE et al (2022) Cochlear implantation in patients with known cognitive impairment: what are the benefits? Otol Neurotol 43(10):1144–1148 36201563 10.1097/MAO.0000000000003701 PMC9649849

[24] Modest MC et al (2015) Cochlear implantation in patients with superficial siderosis: seven cases and systematic review of the literature. Otol Neurotol 36(7):1191–1196 26065403 10.1097/MAO.0000000000000792

[25] Wichova H et al (2022) Cochlear implantation performance outcomes in patients over 80 years old. Laryngoscope Investig Otolaryngol 7(3):847–853 35734051 10.1002/lio2.825 PMC9194979

[26] Wazen JJ et al (2020) Predicting speech outcomes after cochlear implantation in older adults using the self-administered gerocognitive examination test. Otol Neurotol 41(1):e28–e35 31664001 10.1097/MAO.0000000000002425

[27] Young A et al (2024) Long-term cognition and speech recognition outcomes after cochlear implantation in the elderly. Am J Otolaryngol 45(1):104071 37793300 10.1016/j.amjoto.2023.104071

[28] Orçan E, Altınyay Ş, Karamert R (2018) Cochlear implantation in Alzheimer’s disease: a case study. J Hear Sci 8(2):384

[29] Ohemeng KK et al (2019) Cochlear implantation in the old old with cognitive decline. Ear Nose Throat J 98(8):480–481 31035785 10.1177/0145561319846112

[30] Mosnier I et al (2018) Long-term cognitive prognosis of profoundly deaf older adults after hearing rehabilitation using cochlear implants. J Am Geriatr Soc 66(8):1553–1561 30091185 10.1111/jgs.15445

[31] Mosnier I et al (2015) Improvement of cognitive function after cochlear implantation in elderly patients. JAMA Otolaryngol-Head Neck Surg 141(5):442–450 25763680 10.1001/jamaoto.2015.129

[32] Raymond MJ et al (2023) Association of cognitive impairment screening scores with improvements in speech recognition and quality of life after cochlear implantation. JAMA Otolaryngol-Head Neck Surg 149(4):344–351 36729460 10.1001/jamaoto.2022.4825 PMC9896371

[33] Hooper E et al (2022) Systematic review of factors associated with hearing aid use in people living in the community with dementia and age-related hearing loss. J Am Med Dir Assoc 23(10):1669–1675 35988590 10.1016/j.jamda.2022.07.011

[34] Cross H et al (2022) Effectiveness of hearing rehabilitation for care home residents with dementia: a systematic review. J Am Med Dir Assoc 23(3):450-460.e4 34921761 10.1016/j.jamda.2021.11.011

[35] Hooper E et al (2024) Enablers and barriers to hearing aid use in people living with dementia. J Appl Gerontol. 10.1177/07334648231225346 38235997 10.1177/07334648231225346 PMC11168016

[36] Loughrey DG et al (2018) Association of age-related hearing loss with cognitive function, cognitive impairment, and dementia: a systematic review and meta-analysis. JAMA Otolaryngol-Head Neck Surg 144(2):115–126 29222544 10.1001/jamaoto.2017.2513 PMC5824986

[37] Darwich NF, Hwa TP, Ruckenstein MJ (2021) Do patients with dementia benefit from cochlear implantation? Laryngoscope 131(9):1923–1924 33296076 10.1002/lary.29256

[38] Patel D et al (2016) Cognitive decline in the elderly after surgery and anaesthesia: results from the Oxford project to investigate memory and ageing (OPTIMA) cohort. Anaesthesia 71(10):1144–1152 27501155 10.1111/anae.13571 PMC5213281

[39] Rasmussen LS (2006) Postoperative cognitive dysfunction: incidence and prevention. Best Pract Res Clin Anaesthesiol 20(2):315–330 16850780 10.1016/j.bpa.2005.10.011

[40] Theunisse HJ et al (2018) Risk factors for complications in cochlear implant surgery. Eur Arch Otorhinolaryngol 275:895–903 29429025 10.1007/s00405-018-4901-z

[41] Pye A et al (2017) Screening tools for the identification of dementia for adults with age-related acquired hearing or vision impairment: a scoping review. Int Psychogeriatr 29(11):1771–1784 28691649 10.1017/S104161021700120X

[42] Jorgensen LE (2012) The potential impact of undiagnosed hearing loss on the diagnosis of dementia. University of Pittsburgh, Pittsburgh

[43] Dawes P et al (2023) Development and validation of the Montreal cognitive assessment for people with hearing impairment (MoCA-H). J Am Geriatr Soc 71(5):1485–1494 36722180 10.1111/jgs.18241

[44] Völter C et al (2022) Evaluation of the non-auditory neurocognitive test MoCA-HI for hearing-impaired. Front Neurol 13:1022292 36582608 10.3389/fneur.2022.1022292 PMC9792785

[45] Leroi I et al (2022) Support care needs of people with hearing and vision impairment in dementia: a European cross-national perspective. Disabil Rehabil 44(18):5069–5081 34027751 10.1080/09638288.2021.1923071

